# Chronic tophaceous gout with unusual large tophi: case report

**DOI:** 10.11604/pamj.2015.22.132.6447

**Published:** 2015-10-13

**Authors:** Nassira Aradoini, Sofia Talbi, Khadija Berrada, Fatima Zahra Abourazzak, Taoufik Harzy

**Affiliations:** 1 Rheumatology Department, Medical School, Sidi Mohammed Ibn Abdellah University, Hassan II University Hospital, Fez, Morocco

**Keywords:** Gout, tophi, urate crystals

## Abstract

Gout is a metabolic disease, which is characterized by acute or chronic arthritis, and deposition of monosodium urate crystals in joint, bones, soft tissues, and kidneys. But large tophi are unusual in chronic gout. We report the case of a 67-year-oldArabman presenting chronic tophaceous gout with unusual large tophi involving multiple joints: hands, feet, elbows, and knees. Laboratory workup revealed elevated serum uric acid (96 mg/l, normal: 20-74 mg/l), with normal renal function test. In untreated patients, chronic tophaceous gout may develop, which is characterized by chronic destructive polyarticular involvement and tophi. The treatment consists to decrease serum uric acid level which eventually allows the regression of tophi.

## Introduction

Gout is a metabolic disease that can manifest as acute or chronic arthritis, and deposition of monosodium urate crystals in joint, bones and different body tissues, including the skin and soft tissues. Rarely, it can present with tophi as an initial manifestation. Chronic tophaceous gout frequently occurs after 10 years or more of recurrent polyarticular gout. Our case is a rare form of tophaceous gout, which presented with generalized tophi.

## Patient and observation

**Figure 1:**
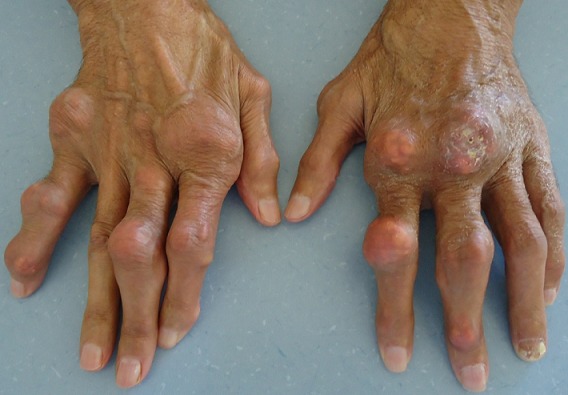
Large tophi over bilateral hands

**Figure 2:**
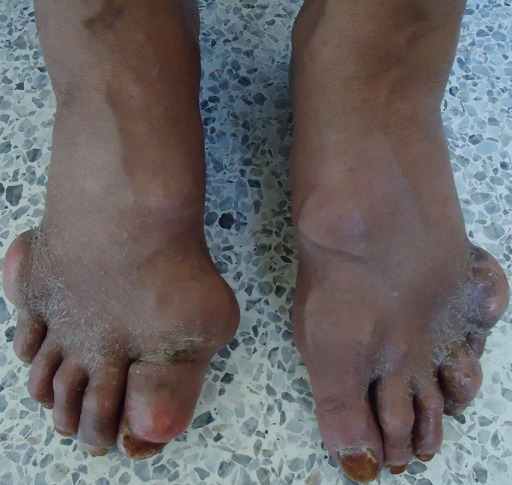
Large tophi over bilateral feet

**Figure 3:**
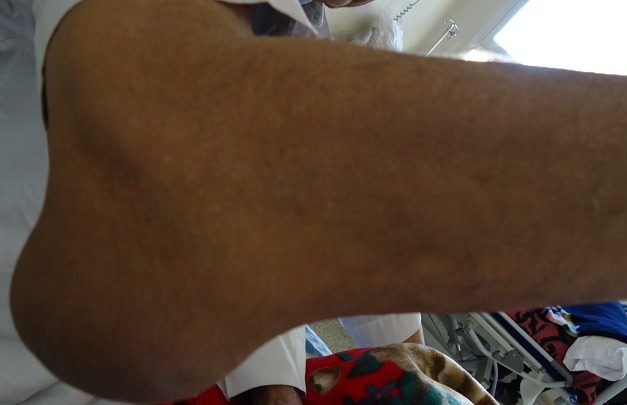
Large tophus of the elbow

**Figure 4:**
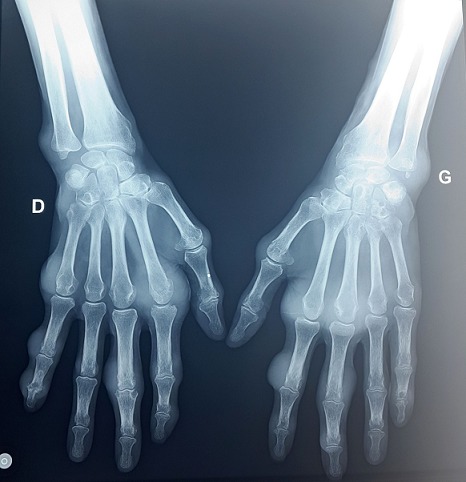
Radiography of both hands showing soft-tissue swelling and periarticular erosions in metacarpophalangeal and interphalangeal joints

**Figure 5:**
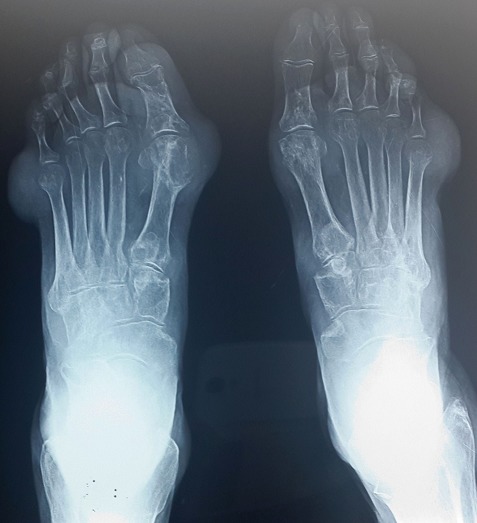
Radiography of the foot showing soft tissue swelling and destruction of the right first metatarsophalangeal joint

A 67-year-old Arab man with an 8-years history of untreated gout was admitted for generalized articular pain. Our patient had only taken traditional medicines for joint pains and did not resort to specific therapy for gout. He was noted to have multiple hard swelling. The swelling developed over 4 years, progressively increasing in size. There was no family history of gout, but personal history of alcohol use, and high purine diet intake. Physical examination revealed that there were multiple large firm tophi over bilateral hands, feet, elbows, and knees (Figure 1, Figure 2, Figure 3). Some of them are ulcerated and discharged white chalky material. He had an average built with BMI of 30,4 and hypertension fortuitously discovered. Laboratory workup revealed elevated serum uric acid (96 mg/l, normal: 20-74 mg/l), with normal renal function test: blood urea 0,4mg/l (normal: 0, 1- 0,5 mg/l) and serum creatinine 10mg/l (normal: 5-18 mg/l). Radiological examination of both hands showed soft-tissue swelling and periarticular erosions in interphalangeal joints (Figure 4). Radiological examination of the feet showed soft tissue swelling and total destruction of the first right metatarsophalangeal joint (Figure 5). Abdominal ultrasonography revealed bilateral caliceal calculi. The patient was treated with Allopurinol (100mg/day) associated to Colchicine (1mg/day). After 3 days, he experienced relief of the joints pain. Then he was referred to urology care for his kidney stones.

## Discussion

Gout is a disorder of purine metabolism and results from long-standing hyperuricaemia and urate crystal deposition in various tissues. In the first stage, it usually affects the first metatarsophalangeal joint and less commonly other joints. The next most frequent localizations are the midtarsi, ankles, knees and arms [1]. Older age, male sex, postmenopausal state and black race are related to a higher risk for development of the disease. Also, the use of certain medications may trigger gout (diuretics, cyclosporine, low doses of aspirin, …) [2]. In untreated patients, chronic tophaceous gout may develop, which is characterized by chronic destructive polyarticular involvement and tophi. Chronic tophaceous gout frequently occurs after 10 years or more of recurrent polyarticular gout. Tophi can occur in soft tissue, osseous tissues, ligaments and different organs and either in presence or absence of gouty arthritis. Tophi are typically found on the helix of the ears, on fingers, toes, wrists and knees, on the olecranon bursae, on the Achilles tendons and also rarely on the sclerae, subconjuctivally [3] and on the cardiac valves [4]. The prevalence of gout is much higher in men than in women and rises with age. Although the prevalence of tophaceous gout, principally the generalized form of it, has decreased in the past years, the disease still exists likely due to the absence of an accurate diagnosis and therapy [5]. Our case is had large tophi, which are unsual in chronic gout. If left untreated, hyperuricemicpatients (serum urate level ≥ 68 mg/l or 400 µmol/l) can evolve from intermittent arthritis to polyarticular tophaceous gout with symptoms between attacks. Lowering serum urate levels with xanthine oxidase inhibitors or uricosuric agents prevents acute flares and tophi development [6]. The recommended target serum uric acid concentration is <60 mg/l (357 µmol/l) [5]. Although controversial, recommendations have been made to achieve a target serum urate level <50 mg/l (297 µmmol/l) in severe chronic gout patients, as this concentration may be associated with greater depletion of synovial fluid crystals and a reduction in tophus size [6–7]. Surgical treatment is seldom required for gout and is usually reserved for cases of recurrent attacks with deformities, severe pain, infection and joint destruction [8]. It's also indicated when tophi are unsightly, painful; or when it interfere with tendon function or causes skin necrosis and ulceration; or encroach upon nerves causing symptoms of compression [9].

## Conclusion

The treatment of gout should be undertaken early in order to avoid the evolution of the disease to the chronic tophaceous form responsible joint deformities and their functional consequences. Our case is a rare form of large tophi complicating untreated chronic gout.
